# Quality Assurance and Patient Safety Measures: A Comparative Longitudinal Analysis

**DOI:** 10.3390/ijerph15081568

**Published:** 2018-07-24

**Authors:** Rafael Manzanera, Diego Moya, Mercedes Guilabert, Manel Plana, Gloria Gálvez, Jordi Ortner, José Joaquín Mira

**Affiliations:** 1 Health and Economic Benefits Department, MC-Mutual, 08037 Barcelona, Spain; dmoya@mc-mutual.com (D.M.); mplana@mc-mutual.com (M.P.); jortner@mc-mutual.com (J.O.); 2 Health Psychology Department, Universidad Miguel Hernández, 03202 Elche, Spain; mguilabert@umh.es; 3 Patient Attention and Social Work Department, Vall d’Hebron University General Hospital, 08035 Barcelona, Spain; ggalvez@vhebron.net; 4 Alicante-Sant Joan Health District, 03013 Alicante, Spain

**Keywords:** quality assurance, patient safety, healthcare organization

## Abstract

Objective: To analyze whether the results on quality assurance and safety culture in a healthcare organization are related to and affected by the actions implemented. Setting: Health Insurance of Work-related Accidents and Occupational Diseases. Methods: The study was conducted as a longitudinal observational study that analyzed the relationship of the Safety Culture and Quality Assurance measurements. Participants who were involved came from small centers with less than eight workers (*N* = 52), big centers (eight and more workers) (*N* = 707), and those centers with quality coordinators (*N* = 91). Data were collected during the years 2015 and 2016. Results: A total of 595 healthcare professionals responded in 2015 and 491 in 2016. The scores showed a positive progression both in Quality Assurance (*T*-test = 3.5, *p* = 0.001) and in Safety Culture (*T*-test = 5.6, *p* < 0.0001). Hence, the gradient of improvement in quality (average 5.5%) was greater compared to that of the safety culture (2.1%). Conclusions: The assessments of the quality assurance goals were consistent with the safety culture assessment. Hence, the results on Safety Culture were observed to be more stable over time.

## 1. Introduction

A commitment on quality objectives is a crucial element of quality policy in high-reliability organizations (HROs), such as hospitals and other healthcare institutions. The quality of care includes objectives related to effectiveness, efficiency, and a patient’s experience [1]. Healthcare organizations are also aware of the importance of promoting safety practices and the resiliency analysis of the clinical practice in order to improve quality. Quality assurance and patient safety are usually analyzed from different approaches that are directly related [2]. The involvement of front-line professionals in safety is a prerequisite for the achievement of an increased quality environment for patients [3]. Healthcare providers’ attitudes, viewpoints, and behaviors in quality, and particularly their safety culture, are crucial for the transformation of healthcare organizations in order to achieve their quality targets, including patients’ satisfaction [4,5,6].

Patient Safety Culture has been defined as the product of individual and group values, attitudes, competencies, and patterns of behavior that determines their commitment, style, and proficiency with the organization’s health and safety programs [7]. The safety culture of a health center offers an indirect means for its involvement in quality [4,8]. Poor involvement of professionals in safety has negative consequences for patients [9].

Safety culture is multidimensional, and usually includes assessment of leadership styles, collaboration and cooperation among staff and front-line professionals, the practice of evidence-based medicine, adequacy of the use of communications channels, a capacity to learn from mistakes, a recognition of errors as system failures rather than individual failures, and a patient-centered approach [10]. These are typically assessed through cross-sectional studies [11].

The patient safety culture measure has been extended in recent years and its impact on results has been analyzed. However, only a few studies have analyzed the relation between safety culture, objective quality [12], and perceived quality by patients [13]. Most of these studies have been carried out through cross-sectional methodologies [11] with the expectation that the results would have implications for directives and professionals in the management of the inherent risks in health activities [8]. However, carrying out measurements may not be enough if it is not accompanied by specific interventions to achieve sustainable changes over time and improvements in patient care.

Measuring quality and safety achievements from healthcare providers’ viewpoints on those aspects of care that need to be improved can contribute to achievement of higher quality care. In this study, a comparison of quality achievements and safety culture measures was conducted in a health organization, in this case, the Mutual Insurance of Work-Related Accidents and Occupational Diseases.

## 2. Methods

Observational, longitudinal study, in which the relationships between safety culture assessments and the evaluation of results of the Quality Assurance Plan of MC Mutual (QA) were analyzed. This study was carried out between May 2015 and November 2016. An annual measure of QA and Safety Culture was obtained. MC Mutual is a Spanish non-profit health organization that provides assistance in case of work-related accidents and occupational diseases for 1.3 million workers. It has 1800 employees, of which 850 are health professionals who serve around 100,000 patients each year. The QA started in 2014 included 18 strategic targets (2 safety targets) grouped into three main areas consisting of the introduction of evidence-based treatment procedures and elaboration of risks maps; the presentation of a report system and quality improvement plans; and explanation of evidence-based safe practices, such as identifying patients correctly, hand-hygiene, the safe use of medicines, and avoiding surgical errors and falls. The targets and actions of the QA can be consulted in QA 2017–2019.

### 2.1. Subjects

In the study of the professionals’ viewpoint of the results of the QA, 143 subjects were invited to respond in 2015 (91 quality assurance coordinators and 52 professionals from centers of less than eight workers (small centers)) and 145 in 2016 (92 quality assurance coordinators and 53 professionals from small centers). These represented approximately a sampling error of 3%, with an expectation that the QA’s acceptance among healthcare professionals would be 60%.

MC Mutual has quality coordinators in all healthcare centers that play a role in connecting their quality activities with the QA. These quality coordinators are either physicians, nurses or physiotherapists who received specific training and were responsible for introducing the QA in their respective centers to their colleagues. The professionals from small centers were selected due to their eligibility in the QA’s implementation for the whole organization with a premise of an easier implementation compared with larger centers, which are usually located in provincial capitals and are closer to intake. There were anticipated differences in the QA’s assessment and Safety Culture since the quality coordinators have participated in the design of activities and thereby have a higher chance to receive feedback on their results. On the other hand, professionals coming from the small centers were a more reliable witness in relation to the level of implementation of the quality and safety strategy.

Eight hundred and fifty professionals (including quality assurance coordinators) in 2015 and 847 in 2016 were invited to respond to the safety culture questionnaire. In 25 cases, the email addresses were found not to be operational. Anonymity was guaranteed and the database did not include personal information.

### 2.2. Materials

The QA evaluation [14] by the professionals included 24 questions. The Safety Culture questionnaire included 10 items explored in two components that explained 60% of the total variance [15] with the following breakdown: attitudinal component (5 items) and instrumental component (5 items). The internal consistency of each factor (Cronbach’s Alpha 0.83 and 0.81, respectively) and the reliability of the questionnaire (intra-class correlation coefficient of 0.87) were analyzed.

The evaluation covered the following areas in both instruments: strategy (inquiry on their commitment to the quality and safety strategy, indicators’ feedback, and risks maps), support systems for clinical decisions (digital record algorithms to make decisions and for accessibility to patient clinical information), equipment (adequacy), follow-up (availability of tests when needed), person-centered care (respect of patients’ values and preferences), evidence-based practice (practices in accordance with guidelines), delays (on scheduled tests, surgery, and outpatient care), and cost-effective treatments (adequacy).

A team composed by two quality technicians and two clinical managers agreed by consensus on the value of the QA actions in terms of degree of implementation (the whole organization versus some centers) and the intensity of the actions that were taken to achieve its implementation across all the centers of MC Mutual (small or large intensity). A scale of 1 to 5 was used, where 1 meant small-range and 5 high-range. This measure ranged between 1 and 25.

### 2.3. Statistics

The percentage of compliance was calculated for each of the items of the QA with respect to the total theoretical maximum score that it can reach (value in the item with respect to the score in the response scale). For the set of elements that made up the areas evaluated, the average compliance was estimated. The same procedure was followed in the case of the Safety Culture measure. By means of a *T*-test for independent samples, the differences between both compliance percentages in the QA and Safety Culture were analyzed.

In addition, a quality assessment was done by one quality technician and one clinical manager that compared the degree of change in the compliance scores with the scope and intensity score of the actions planned in the QA. In this case, the degree of change in the compliance scores in each area was compared with the multi-scope and intensity assessment. Comparisons were classified as high congruence, light congruence, and a lack of congruence. The rank-order correlation using Spearman’s Rho was calculated to measure the association between the degree of change in compliance scores and the scope and intensity scores.

## 3. Results

A total of 96 professionals in 2015 (response rate 67%) and 91 in 2016 (response rate 63%) assessed the results of the QA. In 2015, 70 were quality coordinators and 26 were professionals working in small centers. In 2016, 71 were quality coordinators and 20 were professionals working in small centers. In 2015, 499 professionals responded to the safety culture questionnaire (response rate of 61%) while in 2016 about 400 professionals responded (response rate of 47%). Of these, 62 in 2015 and 62 in 2016 were quality coordinators.

The differences in QA scores between both waves were statistically different, showing an increase in score in the second evaluation of 2016 (*T*-test = 3.5, *p* < 0.001). In the safety culture questionnaire, the total score in the second wave was also greater than that of the first evaluation (*T*-test = 5.6, *p* < 0.0001). The response trends in the evaluation of the QA and Safety Culture results were similar, appreciating that the gradient of the improvement was higher for QA than Safety Culture (Table 1). Moreover, the percentages of change in the case of QA range from 0.3 to 13.4 (average 5.5), while the change in the Safety Culture measure ranges from 0.4 to 8 (average 2.1). The action’s scope and intensity measurements corresponded to the intensity of these changes in seven of the eight cases (Table 2). The Spearman’s Rho value between the degree of change in compliance scores and the scope and intensity scores once ranked was 0.89 (*p* = 0.003).

The scores of quality coordinators were higher than those of professionals, both in the smaller centers (comparisons in QA achievement evaluations) and in the comparison with the assessments of the all professionals (comparisons in Safety Culture) (Table 3).

## 4. Discussion

The trend of the data confirms that the implementation of a strategy in quality and safety has a positive relationship with the results [12]. These analyses have been based on the assessments made by health professionals that indirectly reflect changes in approaches, procedures, and results. It also confirms that the changes in safety culture tend to be slower than those that occur as a result of the implementation of new actions in quality assurance. Other studies have shown that greater benefits are achieved by jointly implementing quality and safety plans [16]. These results, indirectly, support this assertion and highlight that an improvement action in quality has a leverage effect in other relevant areas to achieve safer care for patients.

The improvement actions implemented in this case (definition of a strategy on quality, preparation of a risk map, implementation of an incident notification system, the plan to welcome new professionals, a review of guidelines and protocols or training in quality assurance) have had a positive impact in the evolution of the scores in QA. Leadership is a crucial factor for both: (1) promoting quality actions and a positive perception of quality assurance among professionals; and (2) introducing procedure changes and promoting an adequate work climate that increases patient safety and strengthens performance [17]. In this sense, is expected that job satisfaction and safety culture are related and in recent Spanish research the degree of association between these variables was quantified [18]. In that study, leadership and, specifically, supportive supervision was a key predictor of proactive patient culture.

In this study, we have included the evaluations of quality coordinators of care and the rest of the professionals. These comparisons, as expected, differ in that quality coordinators of care have greater and direct information, so their ratings are slightly more positive than the rest of the professionals. In the same way, it was expected that the evaluations of the professionals of the smaller centers would have the lowest evaluation for two reasons: (1) the information was not disseminated with the same intensity; and (2) the actions started at the larger centers, which resulted in a greater volume of activities. In several studies conducted in a different setting, front-line professionals were found to usually complain more about actions proposed by the directive staff [6]. Also, some studies have shown the sensibility of the safety culture measures to discriminate against the impact of positive actions to improve safety [19] In this case, we observe a similar pattern, although, curiously, there were not greater differences when comparing safety culture measures between quality coordinators and professionals working in smaller centers.

Quality models traditionally measure the scope of actions implemented and this study reinforces the importance of this evaluation criterion. This result could be relevant for directives of healthcare organizations to re-design their assessments of quality and safety policies. While quality results may be appreciated in the short term, safety culture is more stable with time.

Although there is no doubt that quality assurance and patient safety are closely related, there are very few studies that have analyzed empirically the interrelationships between both variables. This study focused on the analysis of the interdependence between both variables in an effort to further improve the efficiency of our measures.

## 5. Limitations

This study is based on subjective measures (on implementation of the QA and Safety Culture Questionnaire). Although the response rates are acceptable, not all professionals responded and the reasons for the non-response were not analyzed. The average scores in QA and safety culture were different, so the improvement was easier in the first than in the second. Quality and safety perceptions are limited to professionals; however, the persons who receive care are patients and their perspectives were not included [20].

## 6. Conclusions

In summary, the systematic participation of professionals assessing the results of the quality plans and safety culture allows us to monitor the degree of deployment and effectiveness of the proposed improvements. While assessments of safety culture inform us of more global attitudinal aspects, the assessments of the implementation of quality plans focus on more specific aspects of direct patient care. The agreement of both measures suggests that the plans in terms of quality and safety achieve the desired level of deployment.

## Figures and Tables

**Table 1:** Response trends and results comparison of the Safety Culture and Quality Assurance measurements assessment.

Areas	QA 2015 (*N* = 96)	QA 2016 (*N* = 91)	QA Improvement ^$^ (%)	Safety Culture 2015 (*N* = 499)	Safety Culture 2016 (*N* = 400)	Safety Culture Improvement * (%)
Strategy	58.2	71.9	13.7 (*p* < 0.0001)	79.7	87.7	8.0 (*p* < 0.0001)
Support systems for clinical decisions	64.9	65.2	0.3 (*p* = 0.921)	92.4	93.5	1.0 (*p* = 0.095)
Equipment	44.0	53.6	4.6 (*p* = 0.226)	86.2	87.4	1.2 (*p* = 0.155)
Follow-up	71.9	75.2	3.3 (*p* = 0.32)	87.4	87.7	0.4 (*p* = 0.636)
Person-centered care	70.6	75.1	4.6 (*p* = 0.061)	82.9	85.8	3.0 (*p* = 0.001)
Evidence-based practice	60.0	71.1	11.1 (*p* < 0.0001)	89.1	89.9	0.7 (*p* = 0.241)
Delays	70.1	74.6	4.5 (*p* = 0.088)	86.9	88.2	1.3 (*p* = 0.065)
Cost-effective treatments	67.8	70.0	2.2 (*p* = 0.535)	86.3	87.9	1.6 (*p* = 0.033)

^$^ Quality Assurance Mutuality Plan of MC Mutual (QA) Improvement is the difference between the QA 2016 and QA 2015 scores. * Safety Culture Improvement is the difference between the Safety Culture 2016 and Safety Culture 2015 scores. *p*-values are the average differences from/in the evaluations in the two QA and safety culture measures.

**Table 2:** Qualitative analysis comparison of the improvements on safety culture and quality assurance measurements and scope and intensity measures of the QA actions implemented.

Areas	QA Improvement ^$^ (%)	Safety Culture Improvement * (%)	Scope × Intensity (Ranged 1 to 25)	Qualitative Assessment	Implementated Actions
Strategy	13.7 ↑↑↑	8.0 ↑↑	20	Greater congruence	QA dissemination and feedback
Support systems for clinical decisions	0.3 =	1.0 =	5	Greater congruence	Digital record
Equipment	4.6 ↑	1.2 =	6	Light congruence	Resuscitation trolleys, gurneys, and other equipment
Follow-up	3.3 ↑	0.4 =	9	Light congruence	Guidelines
Person-centered care	4.6 ↑	3.0 ↑	15	Greater congruence	Surveys to capture patients’ views
Evidence-based practice	11.1 ↑↑↑	0.7 =	12	Lack of congruence	Specific training
Delays	4.5 ↑	1.3 =	9	Light of congruence	Delay criteria stablished
Cost-effective treatments	2.2 =	1.6 =	6	Greater congruence	Diagnosis and treatment criteria defined

^$^ QA Improvement is the difference between the QA 2016 and QA 2015 scores. * Safety Culture Improvement is the difference between the Safety Culture 2016 and Safety Culture 2015 scores. Degree of change in the compliance scores: = 0 to 2.9%, No change; ↑ 3 to 5.9%, Appreciable change; ↑↑ 6 to 8.9%, Important change; ↑↑↑ >9%, Obvious change. Scope × Intensity range: 1 to 12, Small-range; 13 to 19, Neutral-range; 20 to 25 High-range.

**Table 3:** Response trends and results comparisons of the results evaluation of the safety culture and quality assurance measurements of the quality coordinators and the rest of the professionals.

Areas	QA 2015		QA 2016		QA ^$^ Improvement (%)		Safety Culture 2015		Safety Culture 2016		Safety Culture Improvement * (%)	
	Coor ^1^ (*N* = 70)	Prof ^2^ (*N* = 26)	Coor ^1^ (*N* = 71)	Prof ^2^ (*N* = 20)	Coor ^1^	Prof ^2^	Coor ^1^ (*N* = 62)	Prof ^3^ (*N* = 437)	Coor ^1^ (*N* = 62)	Prof ^3^ (*N* = 338)	Coor ^1^	Prof ^3^
Strategy	55.3	63.5	72.4	70.7	17.1 (*p* < 0.0001)	7.2 (*p* = 0.407)	79.6	79.7	90.0	87.2	10.4 (*p <* 0.0001)	7.5 (*p <* 0.0001)
Support systems for clinical decisions	60.4	67.8	65.2	65.5	4.8 (*p* = 0.511)	−2.3 (*p* = 0.456)	94.4	92.2	96.6	92.8	2.2 (*p* = 0.075)	0.6 (*p* = 0.267)
Equipment	45.2	59.2	52.8	56.0	7.6 (*p* = 0.069)	−3.2 (*p* = 0.693)	89.2	85.8	93.0	86.4	3.8 (*p* = 0.042)	0.6 (*p* = 0.504)
Follow-up	73.6	67.4	76.8	69.0	3.2 (*p* = 0.312)	1.6 (*p* = 0.824)	87.8	87.4	93.0	86.8	5.2 (*p* = 0.005)	−0.6 (*p* = 0.507)
Person-centered care	69.6	71.8	76.4	70.0	6.8 (*p* = 0.003)	−1.8 (*p* = 0.538)	83.4	82.8	89.0	85.2	5.6 (*p* = 0.010)	2.4 (*p* = 0.013)
Evidence-based practice	58.2	64.2	70.4	73.0	12.2 (*p* < 0.0001)	8.8 (*p* = 0.189)	89.0	89.2	92.6	89.4	3.6 (*p* = 0.029)	0.2 (*p* = 0.742)
Delays	71.4	67.6	76.1	68.5	4.7 (*p* = 0.051)	0.9 (*p* = 0.860)	88.0	86.8	89.6	88.0	1.6 (*p* = 0.296)	1.2 (*p* = 0.132)
Cost-effective treatments	68.2	66.6	72.0	62.0	3.8 (*p* = 0.312)	−4.6 (*p* = 0.581)	88.6	86.0	92.0	87.2	3.4 (*p* = 0.030)	1.2 (*p* = 0.161)

^1^ Quality coordinators, ^2^ Professionals from centers of less than eight workers, ^3^ All workers from MC Mutual centers, excluding quality coordinators. ^$^ QA Improvement is the difference between the QA 2016 and QA 2015 scores. * Safety Culture Improvement is the difference between the Safety Culture 2016 and Safety Culture 2015 scores. *p*-values are the average differences from every professional group evaluation in every QA and safety culture measure.
